# The Shifts of Diazotrophic Communities in Spring and Summer Associated with Coral *Galaxea astreata, Pavona decussata*, and *Porites lutea*

**DOI:** 10.3389/fmicb.2016.01870

**Published:** 2016-11-22

**Authors:** Yanying Zhang, Qingsong Yang, Juan Ling, Joy D. Van Nostrand, Zhou Shi, Jizhong Zhou, Junde Dong

**Affiliations:** ^1^CAS Key Laboratory of Tropical Marine Bio-resources and Ecology, South China Sea Institute of Oceanology, Chinese Academy of SciencesGuangzhou, China; ^2^Tropical Marine Biological Research Station in Hainan, South China Sea Institute of Oceanology, Chinese Academy of SciencesSanya, China; ^3^Department of Microbiology and Plant Biology, Institute for Environmental Genomics, University of Oklahoma, NormanOK, USA

**Keywords:** diazotrophs, diversity, *nifH*, pyrosequencing, coral reef

## Abstract

The coral holobiont often resides in oligotrophic waters; both coral cells and their symbiotic dinoflagellates possess ammonium assimilation enzymes and potentially benefit from the nitrogen fixation of coral-associated diazotrophs. However, the seasonal dynamics of coral-associated diazotrophs are not well characterized. Here, the seasonal variations of diazotrophic communities associated with three corals, *Galaxea astreata, Pavona decussata*, and *Porites lutea*, were studied using *nifH* gene amplicon pyrosequencing techniques. Our results revealed a great diversity of coral-associated diazotrophs. *nifH* sequences related to *Alphaproteobacteria, Deltaproteobacteria*, and *Gammaproteobacteria* were ubiquitous and dominant in all corals in two seasons. In contrast with the coral *P. decussata*, both *G. astreata* and *P. lutea* showed significant seasonal changes in the diazotrophic communities and *nifH* gene abundance. Variable diazotroph groups accounted for a range from 11 to 49% within individual coral samples. Most of the variable diazotrophic groups from *P. decussata* were species-specific, however, the majority of overlapping variable groups in *G. astreata* and *P. lutea* showed the same seasonal variation characteristics. *Rhodopseudomonas palustris*- and *Gluconacetobacter diazotrophicus*-affiliated sequences were relatively abundant in the summer, whereas a *nifH* sequence related to *Halorhodospira halophila* was relatively abundant in spring *G. astreata* and *P. lutea*. The seasonal variations of all diazotrophic communities were significantly correlated with the seasonal shifts of ammonium and nitrate, suggesting that diazotrophs play an important role in the nitrogen cycle of the coral holobiont.

## Introduction

The coral holobiont is a multi-partner symbiotic system that forms associations with both external and internal microbiota (Mouchka et al., 2010). There is increasing evidence that coral-associated microbial communities are crucial for biogeochemistry and control the health and resilience of coral reef ecosystems (Mouchka et al., 2010; Thompson et al., 2014). Due to their relative size and high per cell activity, a small change in microbial biomass may signal a large reallocation of available energy in the ecosystem (McDole et al., 2012; Haas et al., 2016). Corals often reside in oligotrophic waters. Nitrogen fixation, one of the new fixed nitrogen input into the ecosystem, has long been thought to be an important linchpin to sustain biological productivity in coral reef areas (Wiebe et al., 1975; Capone et al., 1977; Shashar et al., 1994b; Cardini et al., 2014). Nitrogen fixation activity within the coral holobiont has been detected using acetylene reduction and isotopic assays in several coral species (Williams et al., 1987; Shashar et al., 1994a; Lesser et al., 2007; Chimetto et al., 2008; Grover et al., 2014; Benavides et al., 2016). Coral-associated nitrogen-fixing bacteria, capable of fixing and converting gaseous nitrogen (N_2_) to biologically available nitrogen forms, contribute an important source of nitrogen to the coral holobiont (Lesser et al., 2007; Cardini et al., 2014). Both coral cells and their symbiotic dinoflagellates (*Symbiodinium*) have the capacity to assimilate ammonium (Pernice et al., 2012) and benefit from nitrogen-fixing bacteria (Lesser et al., 2007; Cardini et al., 2014; Santos et al., 2014). Additionally, a close relationship was found between the abundance of coral-associated diazotrophs and symbiotic dinoflagellates (Lesser et al., 2007; Olson et al., 2009; Santos et al., 2014), and between coral-associated diazotrophs and coral productivity (Cardini et al., 2014).

Accumulating evidence shows that diazotrophic organisms are ubiquitous members of coral-associated microbial communities and form species-specific associations with their hosts (Lema et al., 2012, 2014b). However, the degree to which nitrogen-fixing bacterial communities are specific to their coral hosts can vary, as both species-specific (Lema et al., 2012) and site-specific (Lema et al., 2014b) nitrogen-fixing microbial communities have been reported. Nitrogen fixation activity in corals is also highly dynamic and can be rapidly affected by changes in environmental conditions (Lesser et al., 2007; Rädecker et al., 2014). It is suggested that coral holobionts harbor both a core microbiome determined by holobiont macroorganisms, and a variable microbiome to adapt to local conditions (Kelly et al., 2014; Ainsworth et al., 2015). As the disturbance of microbial nitrogen-cycling may be tightly linked to coral bleaching and disease (Rädecker et al., 2015), knowledge of the variable diazotrophic groups would help to further evaluate the importance of these communities to the coral host; however, such knowledge is currently lacking.

The *nifH* gene encodes a conserved subunit of the dinitrogenase iron protein responsible for nitrogen fixation, and is conserved in all known diazotrophs (Zehr et al., 2003). As the agreement with 16S rRNA gene-based phylogeny, *nifH* is an ideal molecular target and is widely used for gene-based phylogenetic characterization of diazotrophs (Gaby and Buckley, 2012). In this study, high-throughput Illumina sequencing was used to investigate seasonal and species-specific patterns in diazotrophic communities associated with the corals *Galaxea astreata, Pavona decussata*, and *Porites lutea*. The core and variable diazotrophic microbiome were tested through comparative analysis. The aims were to: (i) investigate the diversity and abundance of diazotrophic communities associated with three coral species; (ii) determine the seasonal shifts of diazotrophic communities and the variable diazotrophic species associated with different coral species; (iii) explore the possible relationship between the coral-associated seasonal-variable diazotroph groups and environmental variables, given the important role of diazotrophic microbes in driving biogeochemical cycles.

## Materials and Methods

### Study Site and Sampling Collection

Coral samples were collected from Luhuitou fringing reef (18°12’19”N, 109°28’27”E) located in Sanya Bay of South China Sea, which is affected by cold-water upwelling during the summer. Cold-water upwelling affects the distribution of a variety of dissolved and particulate forms of nitrogen in Sanya Bay (Huang et al., 2003; Zhang et al., 2010; Wu et al., 2012). Furthermore, in summer, tropical cyclones and monsoonal rainfall may also carry high nutrient loads. Differences in nutrient loads between spring and summer may influence overall diversity of coral-associated diazotrophic communities.

*G. astreata, P. decussata*, and *P. lutea* are important scleractinian species and are all a natural occurrence in the Luhuitou fringing reef region. All coral samples were collected in April and June 2013 using a punch and hammer. Three healthy coral colonies from each coral species were collected at a depth of 5–10 m. Triplicate coral fragments (∼2 cm^2^) for each coral colony were placed in sealed plastic bags, rinsed thoroughly with sterile seawater at the surface, placed on ice and transported to the laboratory (Tropical Marine Biological Research station in Hainan). Samples were cryopreserved at -20°C.

Seawater samples within 20 cm of the coral colonies (*n* = 3) for temperature and salinity were measured using a YSI 6600V2 water quality sonde. Dissolved oxygen (DO) was determined by DO meter, pH was measured using a standard hydrogen electrode and reference electrode, and chemical oxygen demand (COD) was determined by alkaline potassium permanganate method. Inorganic nutrients including nitrate, ammonium, nitrite, and phosphate were measured using standard methods as described previously (Huang et al., 2003).

### DNA Extraction, Amplification, Sequencing, and Data Processing

The coral fragments were suspended in TE buffer and homogenized in a sterilized mortar and pestle with liquid nitrogen. The homogenized solution was transferred to a clean tube and the total community DNA was extracted using an E.Z.N.A.^®^ Soil DNA Kit (Omega Biotek). The DNA was then purified with a Promega Wizard DNA clean-up system (Madison, WI, USA). DNA concentration was measured by Pico Green using a FLUOstar OPTIMA fluorescence plate reader (BMG Labtech, Jena, Germany). The primer sets used to amplify the *nifH* gene were PolF and PolR (Poly et al., 2001) combined with Illumina adapter sequences and barcode sequences (Caporaso et al., 2012). Sample libraries were generated from purified PCR products. The Miseq 300 cycles kit was used for 2x 150 bp paired-end sequencing on Miseq machine (Illumina, San Diego, CA, USA).

Raw *nifH* gene sequences were separated to samples using barcodes and permission of up to one mismatch. Quality trimming was done using Btrim (Kong, 2011). Forward and reverse reads were merged into full-length sequences by FLASH (Magoč and Salzberg, 2011). Sequences were removed if they were too short or contained ambiguous bases. Random re-sampling was performed with 10,000 sequences per sample. The operational taxonomic units (OTUs) were classified using UCLUST at the 90% similarity level, and singletons were removed. The frameshift errors caused by insertions and deletions in DNA sequences were checked and corrected using RDP FrameBot. After processing, the valid *nifH* sequences (310–330 bp) were translated into protein sequences and taxonomic assignment was performed using the RDP FrameBot tool (Wang et al., 2013). The significantly seasonally changed diazotrophic OTUs identified in this study have been deposited in the GenBank database under nucleotide accession numbers KX078090 to KX078212 for *nifH* gene sequences.

### Quantification of *nifH* Gene Copy Number

To quantify the number of copies of the *nifH* gene, the primers PolF and PolR were used. Absolute quantification was carried out on the Lightcycler 480 System (Roche). Standard curves were developed by serially diluting plasmid containing a *nifH* gene to final concentrations from 10^3^ to 10^8^ copies/μl. The qPCR efficiency (E) was 1.90. The *R*^2^ of standards was higher than 0.99. Triple qPCRs were performed for all samples and standard curve concentrations. The specificity of the amplification products was confirmed by melting-curve analysis, and the amplified fragments were checked by electrophoresis in 2% agarose gel to confirm the expected sizes of amplicon. The *nifH* copy number was ultimately expressed as per μg coral colony dry weight.

### Statistical Analysis

All analyses were performed using the R vegan package (R Foundation for Statistical Computing, Vienna, Austria), our R-based pipeline^1^, and the software package CANOCO 4.5 for Windows. Diazotrophic bacteria richness and diversities were calculated using Chao1, Shannon–Wiener’s (*H*’) and evenness. Principal coordinates analysis (PCoA) was used to visualize the changes of overall diazotrophic community structure. Dissimilarity tests by permutational multivariate analysis of variance (PERMANOVA) were performed with Euclidean, Manhattan, Bray–Curtis, and Jaccard for comparing seasonal variation of diazotrophic communities. Significance tests based on unpaired Student’s *t*-test were applied to identify seasonal variation of *nifH* gene diversities and *nifH* copy number abundance. Response ratio analysis was conducted to detect significantly seasonally changed OTU (Deng et al., 2012). Redundancy analysis (RDA) was performed to determine the relationship between variable diazotroph groups and environmental parameters.

## Results

### Environmental Characteristics, Diazotrophic Composition, Community Structure, and *nifH* Gene Abundance

The temperature of ambient seawater was 24.63 ± 0.32°C in the spring, and 25.83 ± 0.45°C in the summer. The average water salinity was 33.46‰ in the spring and 35.01‰ in the summer. The nutrient concentrations of ammonium and phosphorus were higher in the spring. However, the concentration of nitrate was higher in the summer. Higher values of COD and DO were detected in the summer. The concentration of Chlorophyll *a* was higher in the spring (Supplementary Table S1).

After processing, 426,535 high quality *nifH* sequences (310-330 bp) were retrieved from the fragments of coral *G. astreata, Pavona decussata* and *Porites lutea*. Samples were rarefied to 10,000 sequences per sample. All sequences obtained could be assigned to 2,146 OTUs (90% similarity level). The diazotrophic communities were highly diverse (Supplementary Table S2). Shannon–Wiener’s (*H*’) index ranged from 3.24 to 4.43 and Simpson evenness (E) ranged from 0.07 to 0.14. Student’s *t*-test was used to describe the difference between spring and summer samples. The results showed that season did not significantly influence the diazotrophic richness and diversities (*t*-test, *P* > 0.05; Supplementary Table S2).

**FIGURE 1:**
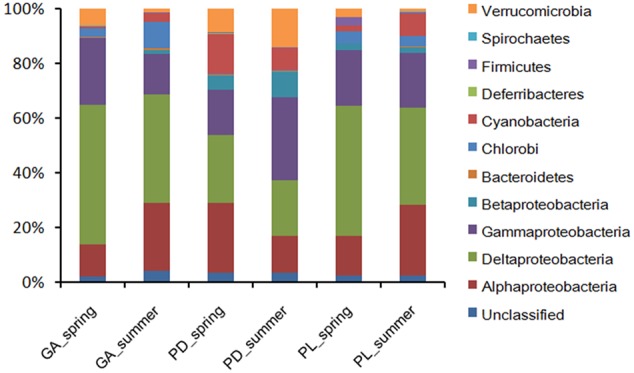
**Diazotrophic composition profiles.** Diazotrophic taxa were categorized at the phylum level except for the *Proteobacteria*, which were categorized by class. GA, *G. astreata*. PD, *P. decussata*. PL, *P. lutea*.

**FIGURE 2:**
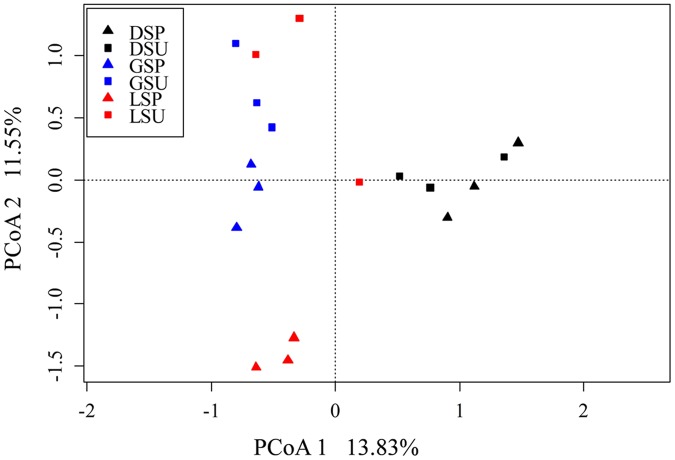
**Principal coordinates analysis (PCoA) of diazotrophic community based on high-throughput *nifH* sequencing data.** The percentage of variation explained by each axis is shown. DSP, spring *P. decussata*. DSU, summer *P. decussata*. GSP, spring *G. astreata*. GSU, summer *G. astreata*. LSP, spring *P. lutea*. LSU, summer *P. lutea*.

The *nifH* sequences retrieved from the three coral species belong to a wide range of bacterial types (**Figure 1**). Although seasonal variations were detected in diazotrophic communities, diazotrophic sequences related to *Alphaproteobacteria, Deltaproteobacteria*, and *Gammaproteobacteria* were ubiquitous and dominant groups that constituted 76.78% of all *nifH* sequences (**Figure 1**). A dissimilarity test based on the Euclidean, Manhattan and Bray–Curtis matrix showed that the diazotrophic communities associated with coral *G. astreata* and *Porites lutea* in spring were significantly different from those in summer (PERMANOVA, *P* = 0.001; **Table 1**), indicating that for these two coral species the diazotrophic communities significantly changed according to the season. However, no significant changes were detected in coral *Pavona decussata*. PCoA also showed the significant seasonal variation in the diazotrophic communities associated with coral *G. astreata* and *Porites lutea* as revealed by the plot. However, *Pavona decussata* -associated diazotrophic communities did not vary in a seasonal manner (**Figure 2**).

**Table 1:** Dissimilarity tests of diazotrophic communities’ dissimilarity between spring and summer by ADONIS.

	Euclidean		Manhattan		Bray–Curtis	
	*F*	*P*	*F*	*P*	*F*	*P*
*Galaxea astreata*	0.279	*0.001*	0.308	*0.001*	1.746	*0.001*
*Pavona decussata*	0.215	0.313	0.258	0.139	1.388	0.117
*Porites lutea*	0.31	*0.001*	0.368	*0.001*	2.332	*0.001*

*Significant differences (*P* < 0.05) are indicated in italics.*

The *nifH* copy number was determined by quantitative PCR with the absolute quantification method. Significant seasonal variations were detected in both *G. astreata* and *Porites lutea* coral colonies (**Table 2**). The *nifH* gene copies in spring *G. astreata* colonies were significantly higher than those in summer (*t*-test, *P* < 0.001). However, the *nifH* gene copies in summer *Porites lutea* colonies were much higher than those collected in spring (*t*-test, *P* < 0.001). No statistical seasonal difference was detected in abundance of *nifH* gene copies from *Pavona decussata* colonies (*t*-test, *P* = 0.841; **Table 2**).

**Table 2:** Abundance of *nifH* copy number from three coral species in spring and summer (expressed as mean value and standard error, SE).

	Spring (*nifH* copy number μg^-1^ colony) Mean ± SE	Summer (*nifH* copy number μg^-1^ colony) Mean ± SE	*P t*-test
*G. astreata*	5.47 ± 1.05	1.34 ± 0.84	<*0.001*
*P. decussata*	1.93 ± 1.71	2.11 ± 1.97	0.841
*P. lutea*	4.07 ± 1.51	151.56 ± 8.25	<*0.001*

*Significant differences (*P* < 0.05) are indicated in italics.*

### Seasonal Variations of Diazotrophic Communities

**FIGURE 3:**
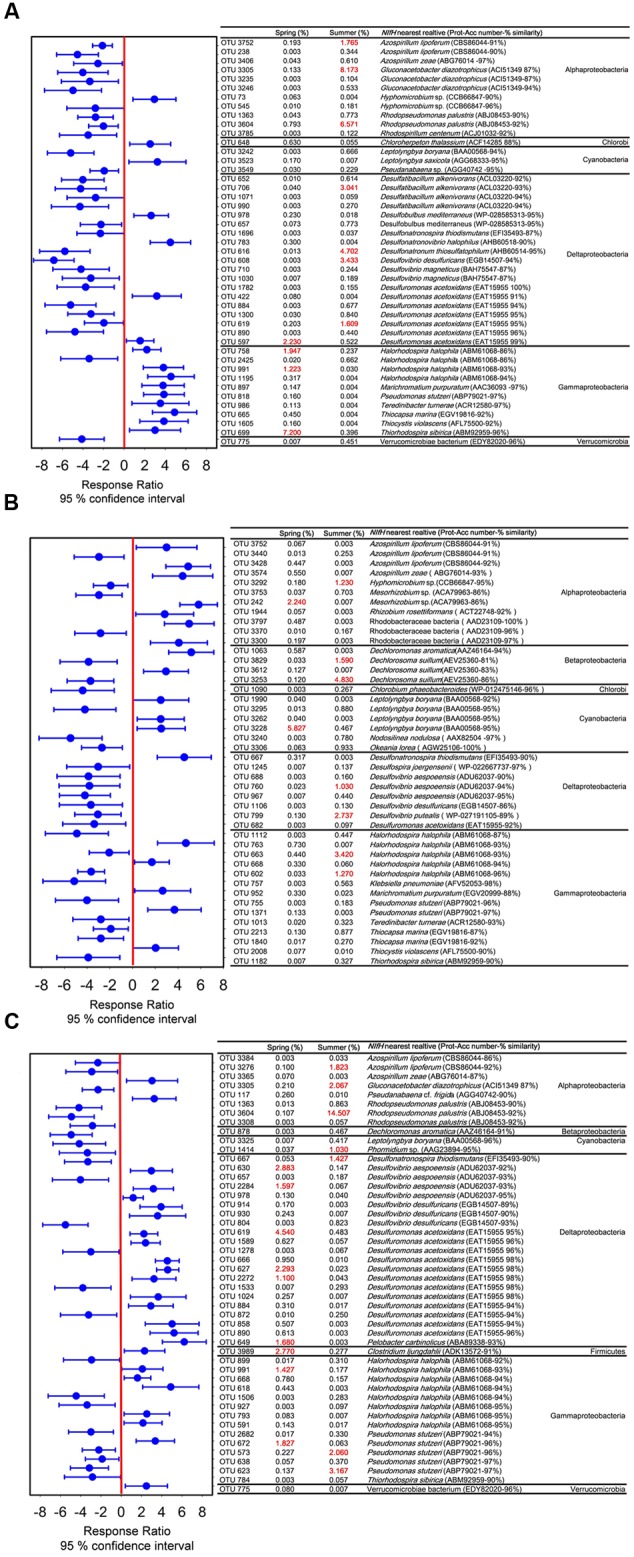
**Significantly seasonal variable OTUs between spring and summer in *G. astreata* (A)**, *P. decussata* **(B)**, and *P. lutea* **(C)** samples determined using the response ratio method at a 95% confidence interval based on the relative abundances. The variable OTUs are represented with their closest sequence match determined from GenBank BLAST, with corresponding accession number and taxonomic affiliation.

Seasonal variable diazotrophic communities were detected at 95% confidence interval. The numbers of variable OTUs were 45, 44, and 48 in coral *G. astreata, Pavona decussata*, and *Porites lutea*, respectively (**Figure 3**). These variable OTUs accounted for a range from 13.92 ± 1.56 to 38.45 ± 5.12% of the total sequences within individual coral samples (Supplementary Table S3). For all coral samples, the majority of variable *nifH* sequences fell within *Alphaproteobacteria, Deltaproteobacteria*, and *Gammaproteobacteria*, and a few within *Cyanobacteria, Betaproteobacteria, Chlorobi, Firmicutes*, and *Verrucomicrobia* (**Figure 3**).

The dominant variable *nifH* sequences retrieved from spring *G. astreata* samples belonged to *Gammaproteobacteria*, however, those dominant in summer *G. astreata* samples belonged to *Alphaproteobacteria* and *Deltaproteobacteria* (**Figure 3A**). Within the *Gammaproteobacteria*, three dominant OTUs were identified (OTU669, OTU991, and OTU758) and constituted 10.37% of the total sequences recovered from spring *G. astreata* samples. The dominant group (OTU669) was affiliated with *Thiorhodospira sibirica* (96% similarity). Both OTU991 and OTU758 were affiliated with *Halorhodospira halophila* (93 and 86% similarity). More than 74% of total summer *G. astreata* variable *nifH* sequences (29.29% of 39.57%) belonged to seven OTUs (OTU3305, OTU3604, OTU3752, OTU706, OTU608, OTU616, and OTU619). The *nifH* sequences within the *Alphaproteobacteria* were relatively abundant in summer. The dominant groups (OTU3305, OTU3604, and OTU3752) comprising up to 16.51% of sequences recovered from summer *G. astreata* samples, were affiliated with *Gluconacetobacter diazotrophicus* (87% similarity), *Rhodopseudomonas palustris* (92% similarity) and *Azospirillum lipoferum* (91% similarity), respectively. The variable OTUs within the *Deltaproteobacteria* were all affiliated with anaerobic sulfate-reducing bacteria of which the dominant members OTU616, OTU608, OTU706 and OTU619 were affiliated with *Desulfonatronum thiosulfatophilum* (95% similarity), *Desulfovibrio desulfuricans* (94% similarity), *Desulfatibacillum alkenivorans* (93% similarity) and *Desulfuromonas acetoxidans* (95% similarity) respectively.

In contrast with *G. astreata* samples, diazotrophic communities associated with *Pavona decussata* samples showed different seasonal variation patterns (**Figure 3B**). OTU242 and OTU3228 dominated the variable *nifH* sequences derived from spring *Pavona decussata* samples. OTU3228 was closely related to a *nifH* sequence from cyanobacterium *Leptolyngbya boryana* (95% similarity). OTU242 demonstrated 86% identity with *Alphaproteobacteria Mesorhizobium* sp. Seven OTUs (OTU799, OTU760, OTU663, OTU602, OTU3292, OTU3253, and OTU3829) constituting more than 65% of total summer *Pavona decussata* variable *nifH* sequences (16.11 of 24.66%) fell within *Alphaproteobacteria, Betaproteobacteria, Deltaproteobacteria*, and *Gammaproteobacteria*. The dominant groups (OTU3253 and OTU3829) represented 6.42% of the total sequences recovered from summer *Pavona decussata* samples affiliated with *nifH* sequences from *Betaproteobacteria Dechlorosoma suillum*. OTU799 and OTU760 were members of the *Deltaproteobacteria* class, affiliated with anaerobic sulfate-reducing bacterial genus *Desulfovibrio*. Within the *Gammaproteobacteria* class, the dominant groups (OTU663 and OTU602) which represented 4.69% of the total sequences recovered from summer *Pavona decussata* samples were both closest to *Halorhodospira halophila*. Furthermore, OTU3292 was affiliated with *nifH* sequences from *Alphaproteobacteria, Hyphomicrobium* sp. (95% similarity).

For coral *Porites lutea, Deltaproteobacteria* dominated the variable *nifH* sequences retrieved from spring samples, and the *Alphaproteobacteria* dominated those from summer ones (**Figure 3C**). Within the *Deltaproteobacteria*, the majority of these sequences were affiliated with the genera *Desulfovibrio* and *Desulfuromonas*, with six dominant ribotypes (OTU619, OTU627, OTU2272, OTU630, OTU2284, and OTU649) comprising up to 14.09% of sequences recovered from spring *Porites lutea* samples. All were affiliated with anaerobic sulfate-reducing bacteria *Desulfovibrio aespoeensis, Desulfuromonas acetoxidans*, and *Pelobacter carbinolicus*. With one exception in *Deltaproteobacteria*, the dominant variable (OTU667) affiliated with *Desulfonatronospira thiodismutans* (90% similarity) was relatively abundant in spring *Porites lutea* samples. Three dominant ribotypes (OTU3604, OTU3305, and OTU3276) falling within the *Alphaproteobacteria*, constituted 18.39% of total summer *Porites lutea nifH* sequences. The most dominant group (OTU3604), which represented 14.51% of the total sequences recovered from summer *Porites lutea* samples, affiliated with purple non-sulfur bacterium *Rhodopseudomonas palustris* (92% similarity). Two other *Alphaproteobacteria* groups (OTU3305 and OTU3276) affiliated with *Gluconacetobacter diazotrophicus* (87% similarity) and *Azospirillum lipoferum* (92% similarity), respectively. There were four dominant variable ribotypes recovered from *Porites lutea* samples falling within the *Gammaproteobacteria*. Two ribotypes (OTU991 and OTU672) were relatively abundant in spring samples affiliated with *Halorhodospira halophila* (93% similarity) and *Pseudomonas stutzeri* (96% similarity), respectively. Other groups (OTU623 and OTU573) were significantly abundant in summer samples, and both affiliated with *Pseudomonas stutzeri* (96–97% similarity). Within the *Cyanobacteria*, the dominant group (OTU1414), affiliated with *Phormidium* sp. (95% similarity), was relatively abundant in summer *Porites lutea* samples. In addition, the dominant ribotype (OTU3989) affiliated with *Firmicutes, Clostridium arbusti* (93% similarity), was relatively abundant in spring *Porites lutea* samples.

The majority of variable *nifH* groups from coral *Pavona decussata* were species-specific. Only one overlap variable OTU was found between the coral *G. astreata* and *Pavona decussata* samples and two overlap OTUs were detected between the coral *Pavona decussata* and *Porites lutea* colonies (Supplementary Table S4). These overlap OTUs were generally present in relatively low abundance in all *Pavona decussata* colonies. Ten overlap OTUs were recovered from both *G. astreata* and *Pavona decussata*, of which the majority of dominant groups showed the same seasonal variation (Supplementary Table S4). Two *Alphaproteobacteria* OTUs (OTU3604 and OTU3305) were relatively abundant, representing 14.74 and 16.57% of the total sequences recovered from summer *G. astreata* and *Porites lutea*, respectively. The *Gammaproteobacteria* variable group (OTU991) was dominant in both *G. astreata* and *Porites lutea* spring colonies. One exception to this pattern is the *Deltaproteobacteria* variable (OTU619), which dominated summer *G. astreata* samples and spring *Porites lutea* samples.

### The Relationship between Seasonal Variable Diazotrophic Community and the Surrounding Seawater Environmental Factors

**FIGURE 4:**
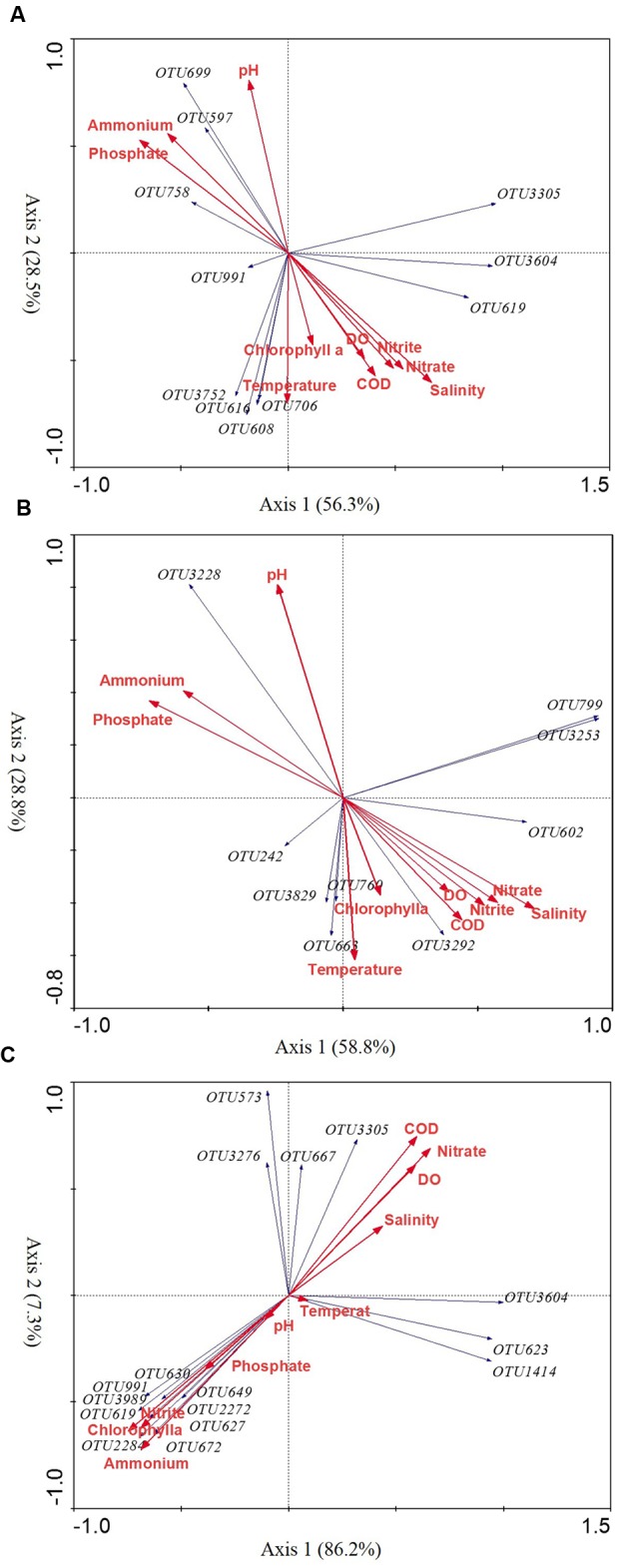
**The redundancy analysis (RDA) ordination biplot showing the relationship between environmental variables and significantly seasonal variable OTUs in *G. astreata* (A)**, *P. decussata* **(B)**, and *P. lutea* **(C)** samples. Only abundant variable OTUs (1%) are shown in the biplot.

To explore the possible relationship between the seasonal variable diazotrophic microbial community and environmental variables, RDA was performed. The results showed that all seasonal variable diazotrophic communities of three coral species were significantly correlated with ammonium and nitrate (*P* < 0.05, **Table 3**). In addition, the seasonal variable diazotrophic communities of coral *G. astreata* and *Pavona decussata* were significantly correlated with phosphorus (*P* < 0.05, **Table 3**). Axis 1 and 2 of the RDA biplot together were shown to contribute 84.8, 87.6, and 93.5% to the overall pattern of three coral species, respectively (**Figure 4**). Most of the dominant variable diazotrophic OTUs of spring samples were highly positively correlated with ammonium and phosphate (**Figure 4**). In contrast, those from summer samples were negatively correlated with ammonium and phosphate, but positively correlated with nitrate, salinity and temperature (**Figure 4**).

**Table 3:** Monte Carlo permutation test of environmental attributes with *nifH* high-throughput sequencing data.

	*G. astreata*	*P. decussata*	*P. lutea*
Ammonium	*0.009*	*0.045*	*0.044*
Nitrate	*0.005*	*0.023*	*0.048*
Nitrite	0.061	0.083	0.092
Phosphate	*0.013*	*0.022*	0.661
Chlorophyll *a*	0.296	0.223	*0.026*
pH	0.076	0.077	0.951
COD	0.052	0.072	0.099
DO	0.083	0.07	0.224
Salinity	*0.045*	*0.020*	0.396
Temperature	*0.018*	*0.047*	0.995

*Significant differences (*P* < 0.05) are indicated in italics.*

## Discussion

Although high-throughput sequencing approaches are now commonly applied to investigate coral-associated microbial communities (Ceh et al., 2011; Chen et al., 2011; Lee et al., 2012; Mckew et al., 2012; Sunagawa et al., 2009), it has not been widely used to target nitrogen-fixing functional genes (*nifH*) to explore the coral-associated diazotrophic communities (Lema et al., 2014b). Compared with previous high-throughput sequencing studies on diazotrophic communities associated with the common Great Barrier Reef (GBR) coral *Acropora millepora* (Lema et al., 2014b), a much greater diversity of bacteria having the potential to fix nitrogen (possess the *nifH* gene) associated with *G. astreata, Pavona decussata*, and *Porites lutea* from Luhuitou fringing reef, South China Sea (**Table 1**), and might be attributed to the differences in coral species. *Alphaproteobacteria* was reported as the dominant diazotrophic group associated with the common GBR coral *Acropora* (i.e., *A. millepora* and *A. muricata*) (Lema et al., 2012), while *Gammaproteobacteria* was the most commonly found class in the Hawaiian *Montipora* corals (*M. capitata* and *M. flabellata*) (Olson et al., 2009). In the present study, *nifH* sequences related to *Alphaproteobacteria, Deltaproteobacteria*, and *Gammaproteobacteria* were the ubiquitous and dominant groups of three coral species in two seasons. These three groups constitute from 46.65 to 91.1% of the sequences within individual coral samples (**Figure 1**), suggesting that those groups play an important functional role in the coral holobiont. The coral holobiont is a complex habitat, and microenvironmental variability can strongly influence the abundance of associated microbial communities (Rohwer et al., 2002; Ainsworth et al., 2010). Previous results reported that the coral-associated bacterial community showed rapid seasonal changes for several coral species distributed throughout different regions (Ceh et al., 2011; Chen et al., 2011). However, season did not influence the diazotrophic communities associated with common GBR coral *A. millepora* (Lema et al., 2014b). Significant differences were detected in the diazotrophic abundance of *G. astreata* and *Porites lutea*, indicating that for these two coral species the diazotrophic communities significantly changed according to the season.

Understanding the seasonal dynamics of this association is important, as they have ecological implications. In this study, seasonal variable diazotrophic sequences were detected based on the relative abundances at 95% confidence interval. The analyses showed that three corals have a low diversity group of seasonal variable species (range from 44 to 48 OTUs) whose abundance varies widely across individuals (Supplementary Table S3). These results suggested that there are core diazotrophic microbiomes associated with corals and these cores are complemented with seasonal variable diazotrophic microbiomes. Most of the variable *nifH* groups from *Pavona decussata* were species-specific. Only a few variable OTUs overlap with two other coral species. This may be an indicator of a specific association between coral and diazotrophic microorganisms. The majority of overlapping OTUs in *G. astreata* and *Porites lutea* showed the same seasonal variation suggesting that except host species, the variation of diazotrophic communities might be related to the biogeochemical cycling processes within the holobiont, given the important role of diazotrophs in driving biogeochemical cycles (Rädecker et al., 2014).

*Alphaproteobacteria* affiliated with the order *Rhizobiales* were reported as the continuous and dominant diazotrophic assemblages associated with the common GBR *Acropora* corals (Lema et al., 2012, 2014a,b). Additionally, the rhizobia found within pure mucus samples of New Caledonia reefs (predominantly composed of *Acropora* sp.) was much higher than in the surrounding seawater in the summer period and 400-fold higher in the winter (Camps et al., 2016). In the present study, the variable *Alphaproteobacteria* affiliated with *Rhizobiales* and *Rhodospirillales*. One rhizobial group (OTU3604) was most closely affiliated with purple non-sulfur phototrophic bacterium *Rhodopseudomonas palustris*. This group dominated all spring *G. astreata* and *Porites lutea* colonies, representing up to 6.57 and 14.51% of the total *nifH* sequences in spring *G. astreata* and *Porites lutea* samples, respectively (**Figure 3**). The *Rhodospirillales* groups closely related to *Gluconacetobacter diazotrophicus* and *Azospirillum lipoferum* were also the dominant variable groups found in all *G. astreata* and *Porites lutea* colonies. It is notable that the most dominant variable group of *G. astreata* was closely related to *Gluconacetobacter diazotrophicus*. Here *Rhodopseudomonas palustris*- and *Gluconacetobacter diazotrophicus*-affiliated OTUs were not correlated with investigated environmental factors, which suggested the seasonal variations of these diazotrophs are not affected by environmental factors. *Rhodopseudomonas palustris* is notable for its ability to flexibly switch between four different modes of metabolism: photoautotrophic, photoheterotrophic, chemoautotrophic, and chemoheterotrophic (Larimer et al., 2004), which might protect it from high concentrations of oxygen arising from dinoflagellate photosynthesis in coral tissues (Kuhl et al., 1995). The most common physiological characteristics of *Gluconacetobacter diazotrophicus* are its growth and nitrogen fixation at low pH and that nitrogen fixation is not affected by high concentrations of nitrate nitrogen (Pedraza, 2008; Eskin et al., 2014). Additionally, it is capable of undergoing nitrogen fixation with low amounts of ammonium-based nitrogen (Stephan et al., 1991; Fisher and Newton, 2005). In contrast with *G. astreata* and *Porites lutea*, the dominant *Alphaproteobacteria* variable groups in *Pavona decussata* showed different patterns. One rhizobial group affiliated with *Hyphomicrobium* sp. was the dominant group in spring colonies while another rhizobial group affiliated with *Mesorhizobium* sp. was the dominant group in summer colonies.

The second most seasonal variable group of diazotrophs detected by our *nifH*-based gene assay were all affiliated with anaerobic sulfate-reducing *Deltaproteobacteria*. Anaerobic sulfate-reducing bacteria were commonly detected in both 16S rRNA and *nifH* gene surveys of presumably healthy corals (Olson et al., 2009; Sunagawa et al., 2010 Lema et al., 2012, 2014b; Fernando et al., 2015). Although oxygenic photosynthesis renders most of the coral interior oxic during the day, coral microhabitats may host diazotrophic communities under oxygen-depleted conditions within the gastrodermis (Hoegh-Guldberg and Williamson, 1999) and microaerophilic regions in the gastrovascular cavities of coral polyps (Agostini et al., 2012). Additionally, coral tissues compromised by contact with stagnant water or sediment and coral surfaces (mucus layer) and skeletons enable anaerobic forms of bacterial respiration and fermentation to occur within the holobiont (Thompson et al., 2014). The present study results showed that the seasonal variations of *Deltaproteobacteria* diazotrophs differed among coral species. The *Deltaproteobacteria* groups were relatively abundant in summer *G. astreata* and *Pavona decussata* samples and spring *Porites lutea* samples. The dominant variable *Deltaproteobacteria* groups of *G. astreata* and *Pavona decussata* were positively correlated with nitrate and salinity; however, the dominant variable *Deltaproteobacteria* groups of *Porites lutea* were negatively correlated with nitrate, but positively correlated with ammonium (**Figure 4**). Additionally, the dominant variable *Deltaproteobacteria* groups also varied among coral host species, indicating that both host species and environmental factors cause seasonal shifts of *Deltaproteobacteria* diazotrophs.

*Gammaproteobacteria* genus *Vibrio* was reported as consistent members of diazotrophic assemblages associated with Hawaiian corals in the genus *Montipora* (Olson et al., 2009) and common GBR coral *A. millepora* (Lema et al., 2014b) based on *nifH* sequencing. Here, genus *Vibrio* were also consistent members of diazotrophic assemblages associated with three coral species, and no significant seasonal variation was detected based on *nifH* sequence. However, genus *Vibrio* is a variable bacterial group associated with coral *G. astreata* and *Porites lutea* and *Vibrio*-affiliated bacterial sequences represented 0–0.84% of the 16S rRNA gene sequences recovered. The dominant seasonal variable *Gammaproteobacteria* affiliated with *Halorhodospira halophila, Thiorhodospira sibirica*, and *Pseudomonas stutzeri*. *Halorhodospira halophila*, an obligately photosynthetic and extremely halophilic purple sulfur bacterium (Tsuihiji et al., 2006), showed relative abundance in spring coral *G. astreata* and *Porites lutea*, but was dominant in summer *Pavona decussata* samples. One group affiliated with *Thiorhodospira sibirica*, one of the alkaliphilic purple sulfur bacteria (Bryantseva et al., 1999), represented up to 7.2% of the total *nifH* sequences in spring *G. astreata* samples and positively correlated with ammonium and phosphate. Additionally, *Pseudomonas stutzeri*, a nitrogen-fixing and denitrifying bacterium (Lalucat et al., 2006), was relatively abundant in summer *Porites lutea* samples.

The most dominant variable group of *Pavona decussata*, closely related to *Cyanobacteria Leptolyngbya boryana*, represented up to 5.83% of the total *nifH* sequences in spring *Pavona decussata* samples, positively correlated with ammonium and phosphate. Another *Cyanobacteria* group closely related to *Phormidium* sp. was relatively abundant in summer *Porites lutea* samples. Both genera *Leptolyngbya* and *Phormidium* have previously been reported in a number of different coral species black band disease (BBD) samples where they have been hypothesized to play roles as pathogens (Frias-Lopez et al., 2003; Richardson and Kuta, 2003; Myers et al., 2007). The *nifH* phylotypes affiliated with *Firmicutes Clostridium ljungdahlii* were only detected showing significant seasonal variation in *Porites lutea* samples, comprising 2.77% of *nifH* phylotypes derived from spring *Porites lutea* and positively correlated with ammonium and phosphate.

The seasonal variations in coral-associated bacterial and diazotrophic communities may be influenced by the nutrient loads between spring and summer. The sample collection location Sanya Bay is affected by cold-water upwelling during the summer (Huang et al., 2003; Zhang et al., 2010; Wu et al., 2012). Cold-water upwelling affects the distribution of a variety of dissolved and particulate forms of nitrogen in Sanya Bay (Huang et al., 2003). Ammonium was the predominant dissolved nitrogen in spring, while nitrate was the predominant dissolved nitrogen in summer (Supplementary Table S1). In our previous study, the functional gene composition of the microbial community was significantly correlated with the concentrations of inorganic nitrogen and phosphate (Zhang et al., 2015). Here, all diazotrophic communities of the three coral species were significantly correlated with ammonium and nitrate (**Table 3**). In addition, the diazotrophic communities of coral *G. astreata* and *Pavona decussata* were significantly correlated with phosphorus (**Table 3**). This suggested that the apparent seasonal change in diazotrophic communities of corals could be linked to the seasonal shifts of nutrients. A priority for future studies should be to identify environmental variables contributing to these shifts in coral bacterial communities and to determine how they influence the health of the coral host.

## Conclusion

This study revealed a much greater diversity of diazotrophs associated with *G. astreata, Pavona decussata*, and *Porites lutea*. *Alphaproteobacteria, Deltaproteobacteria*, and *Gammaproteobacteria* were the ubiquitous and dominant groups in all corals in two seasons. Seasonal factors did not cause shifts in diazotrophic richness and diversities of the three coral species; even no shifts in diazotrophic communities and abundance were observed in coral *Pavona decussata*. In contrast, the diazotrophic communities and *nifH* gene abundance of both *G. astreata* and *Porites lutea* showed significant seasonal changes. Most of the variable *nifH* groups from *Pavona decussata* were species-specific. The dominant overlap OTUs in *G. astreata* and *Porites lutea* showed the same seasonal variation. The seasonal variations of diazotrophic communities were significantly correlated with the seasonal shifts of nutrients. Variable diazotroph groups are widely distributed in the environment and may be of relevance to diverse metabolic potential, such as carbon fixation and sulfate reduction. This suggests that their potential to provide additional sources of fixed nitrogen to the coral holobiont may be functionally important. However, almost all metabolic potential of these diazotrophs was referred from crops and land plants. The physiological roles of these nitrogen-fixing symbionts in the nitrogen budget and cycling within corals need to be investigated in detail.

## Author Contributions

YZ and JD conceived the research. YZ and QY performed the experiments. YZ wrote the manuscript. JVN and JZ edited the manuscript. QY and ZS contributed sampling or data analysis pipeline. All authors reviewed and accepted the manuscript.

## Conflict of Interest Statement

The authors declare that the research was conducted in the absence of any commercial or financial relationships that could be construed as a potential conflict of interest.

## Footnotes

1 http://ieg.ou.edu/microarray/
