# Supplementary material for: The Shifts of Diazotrophic Communities in Spring and Summer Associated with Coral Galaxea astreata, Pavona decussata, and Porites lutea
Source: Front Microbiol. 2016 Nov 22;7:1870. doi: 10.3389/fmicb.2016.01870 (PMC5118425; doi:10.3389/fmicb.2016.01870)
Supplement: Supplementary file 1 [file Table_1.DOC]

Table S1 Environmental parameters of seawater around the coral samples (Expressed as mean value and standard error, mean±SE).

|  | Spring  Mean±SE. | Summer  Mean±SE |
| --- | --- | --- |
| Temperature (℃) | 24.63±0.32 | 25.83±0.45 |
| Salinity (‰) | 33.46±0.31 | 35.01±0.09 |
| pH | 8.12±0.01 | 8.11±0.01 |
| Ammonium (µg L^-1^) | 36.92±0.57 | 12.95±0.12 |
| Nitrate (µg L^-1^) | 17.93±1.53 | 55.70±0.13 |
| Nitrite (µg L^-1^ ) | 2.28±0.38 | 2.25±0.06 |
| Phosphate (µg L^-1^) | 12.18±0.88 | 5.30±0.66 |
| Chlorophyll a (µg L^-1^) | 1.81±0.19 | 1.43±0.11 |
| COD (mg L^-1^) | 0.25±0.01 | 1.53±0.09 |
| DO (mg L^-1^) | 6.03±0.05 | 6.96±0.05 |
