# Supplementary material for: The Shifts of Diazotrophic Communities in Spring and Summer Associated with Coral Galaxea astreata, Pavona decussata, and Porites lutea
Source: Front Microbiol. 2016 Nov 22;7:1870. doi: 10.3389/fmicb.2016.01870 (PMC5118425; doi:10.3389/fmicb.2016.01870)
Supplement: Supplementary file 2 [file Table_2.DOC]

Table S2 The richness and diversity information of *nifH* sequences from three coral species (n=3) in two seasons. (Expressed as mean value and standard error, mean±SE).

|  | *G. astreata* | | | *P. decussata* | | | *P. lutea* | | |
| --- | --- | --- | --- | --- | --- | --- | --- | --- | --- |
|  | spring | summer | P | spring | summer | P | spring | summer | P |
|  | (mean±SE) | | t-test | (mean±SE) | | t-test | (mean±SE) | | t-test |
| Chao 1 | 406±76 | 220±120 | 0.087 | 551±329 | 199±91 | 0.148 | 408±96 | 273±142 | 0.246 |
| OTUs | 298±64 | 181±91 | 0.14 | 415±227 | 127±53 | 0.1 | 317±75 | 191±97 | 0.149 |
| Shannon (H’) | 4.43±0.31 | 3.74±0.74 | 0.212 | 4.17±0.64 | 3.24±0.30 | 0.085 | 4.34±0.31 | 3.71±0.52 | 0.156 |
| Simpson E | 0.14±0.08 | 0.14±0.04 | 0.951 | 0.07±0.03 | 0.12±0.06 | 0.299 | 0.12±0.06 | 0.13±0.10 | 0.891 |
