# Supplementary material for: The Shifts of Diazotrophic Communities in Spring and Summer Associated with Coral Galaxea astreata, Pavona decussata, and Porites lutea
Source: Front Microbiol. 2016 Nov 22;7:1870. doi: 10.3389/fmicb.2016.01870 (PMC5118425; doi:10.3389/fmicb.2016.01870)
Supplement: Supplementary file 3 [file Table_3.DOC]

Table S3 The percentage of the diazotrophic variable OTUs accounted for the total sequences (Expressed as mean value and standard error, mean±SE).

|  | spring | summer |
| --- | --- | --- |
| *G. astreata* | 17.11±3.97 | 38.45±5.12 |
| *P. decussata* | 13.92±1.56 | 24.85±3.79 |
| *P. lutea* | 26.81±1.84 | 32.62±8.11 |
