# Supplementary material for: The Shifts of Diazotrophic Communities in Spring and Summer Associated with Coral Galaxea astreata, Pavona decussata, and Porites lutea
Source: Front Microbiol. 2016 Nov 22;7:1870. doi: 10.3389/fmicb.2016.01870 (PMC5118425; doi:10.3389/fmicb.2016.01870)
Supplement: Supplementary file 4 [file Table_4.DOC]

Table S4 The overlap of variable diazotrophic communities among three coral species.

|  | ***G. astreata*** | | ***P. decussata*** | | ***P. lutea*** | |  |  |
| --- | --- | --- | --- | --- | --- | --- | --- | --- |
|  | **Spring (%)** | **Summer (%)** | **Spring (%)** | **Summer (%)** | **Spring (%)** | **Summer (%)** | ***NIfH* nearest relative (Prot-Acc number-% similarity)** |  |
| OTU1363 | 0.043 | 0.773 | / | / | 0.013 | 0.863 | *Rhodopseudomonas palustris* (ABJ08453-90%) | Alphaproteobacteria |
| OTU3604 | 0.793 | **6.571** | / | / | 0.107 | **14.507** | *Rhodopseudomonas palustris* (ABJ08453-92%) | Alphaproteobacteria |
| OTU3305 | 0.133 | **8.173** | / | / | 0.21 | **2.067** | *Gluconacetobacter diazotrophicus* (ACI51349 -87%) | Alphaproteobacteria |
| OTU3752 | 0.193 | **1.765** | 0.067 | 0.003 | / | / | *Azospirillum lipoferum* (CBS86044-91%) | Alphaproteobacteria |
| OTU657 | 0.073 | 0.773 | / | / | 0.003 | 0.187 | *Desulfovibrio aespoeensis* (ADU62037-93%) | Deltaproteobacteria |
| OTU978 | 0.23 | 0.018 | / | / | 0.13 | 0.04 | *Desulfovibrio aespoeensis* (ADU62037-95%) | Deltaproteobacteria |
| OTU890 | 0.003 | 0.44 | / | / | 0.613 | 0.003 | *Desulfuromonas acetoxidans* (EAT15955-96%) | Deltaproteobacteria |
| OTU884 | 0.003 | 0.677 | / | / | 0.31 | 0.017 | *Desulfuromonas acetoxidans* (EAT15955-94%) | Deltaproteobacteria |
| OTU619 | 0.203 | **1.609** | / | / | **4.54** | 0.483 | *Desulfuromonas acetoxidans* (EAT15955- 95%) | Deltaproteobacteria |
| OTU667 | / | / | 0.317 | 0.003 | 0.053 | **1.427** | *Desulfonatronospira thiodismutans* (EFI35493-90%) | Deltaproteobacteria |
| OTU668 | / | / | 0.33 | 0.06 | 0.78 | 0.157 | *Halorhodospira halophila* (ABM61068-94%) | Gammaproteobacteria |
| OTU991 | **1.223** | 0.03 | / | / | **1.427** | 0.177 | *Halorhodospira halophila* (ABM61068-93%) | Gammaproteobacteria |
| OTU775 | 0.007 | 0.451 | / | / | 0.08 | 0.007 | Verrucomicrobiae bacterium (EDY82020-96%) | Verrucomicrobia |
